# The Effect of Alpha-Tocopherol on the Expression of Epidermal Growth Factor Receptor and Transforming Growth Factor Beta Genes in Three Developmental Stages of *Echinococcus granulosus*

**Published:** 2020

**Authors:** Seyyed Jafar NOSRATABADI, Nasim HAYATI ROODBARI, Mohammad Hossein MODARRESI, Alireza FARSINEJAD, Majid FASIHI HARANDI

**Affiliations:** 1. Department of Biology, School of Basic Sciences, Science and Research Branch, Islamic Azad University, Tehran, Iran; 2. Department of Medical Genetics, School of Medicine, Tehran University of Medical Sciences, Tehran, Iran; 3. Department of Laboratory Hematology and Blood Banking, Faculty of Allied Medicine, Kerman University of Medical Sciences, Kerman, Iran; 4. Research Center for Hydatid Disease in Iran, School of Medicine, Kerman University of Medical Sciences, Kerman, Iran

**Keywords:** Alpha-tocopherol, In vitro, Hydatid disease, Epidermal growth factor receptor (EGFR), Transforming growth factor-beta (TGF- β)

## Abstract

**Background:**

In recent decades platyhelminths have been used as model organisms to address some of the fundamental questions related to the growth and development of animal organisms. Epidermal Growth Factor Receptors (EGFR) and Transforming Growth Factor beta (TGF-beta) have a regulatory role in the growth and development of *Echinococcus* species. This study determined the effect of alpha-tocopherol on the expression of *EGFR* and *TGF*-beta genes in three in vitro developmental stages of *E. granulosus*.

**Methods:**

*E. granulosus* protoscoleces were cultured in diphasic medium containing bovine serum and CMRL 1066. Three developmental stages of *E. granulosus*, i.e. invaginated protoscoleces, evaginated protoscoleces and three-proglottid worms, were treated by alpha-tocopherol (250 μg/ml for 36 h) and the expression of *EGFR* and *TGF*-beta genes were evaluated by using qPCR analysis.

**Results:**

Intact protoscoleces were successfully developed to the segmented worms in diphasic culture media. Higher levels of both *EGFR* and *TGF*-beta gene expression were observed in the invaginated protoscoleces as well as the segmented worms in comparison to the non-treated controls.

**Conclusion:**

Administration of alpha-tocopherol to different developmental stages of *E. granulosus* significantly enhanced *EGFR* and *TGF*-beta expression in the parasite*.* Both oxidant and non-oxidant activities of alpha-tocopherol could explain the study findings. Overexpression of the genes could in turn enhance growth factor effects and facilitates the viability of the parasite.

## Introduction

Major advances have been made in our knowledge of the molecular and cellular biology of invertebrates using different animal model systems (1). In recent decades using helminths as model organisms, some of the fundamental questions related to the biology and development of animal organisms have been addressed. Among platyhelminths, the tapeworms (Cestoda) are a group of parasitic helminths with complex life cycles involving definitive and intermediate hosts. Several cestode species have been selected for different in vivo and in vitro studies, including species within genus *Taenia*, *Echinococcus* and *Hymenolepis*. *Taenia crassiceps* (2), *T. solium* (3), *Hymenolepis diminuta* (4), *Mesocestoides corti* (5), as well as different *Echinococcus* species.

*Echinococcus granulosus* is a zoonotic helminth in Taeniidae family with a worldwide distribution. The adult worms mature in the small intestine of canid definitive hosts and the larval stage (metacestode), which is a fluid-filled cyst containing larval organisms called protoscoleces, lives in the visceral organs of the intermediate hosts, including herbivorous or omnivorous livestock. Cystic echinococcosis caused by the metacestode stage, has been shown to impose major economic and medical burdens on human and animals (6). Understanding the cellular and molecular basis of the growth, development and proliferation of the parasite is essential for reducing burden and disease control.

Growth and development in *Echinococcus* are regulated by Epidermal Growth Factor (EGF) as well as Transforming Growth Factor beta (TGF-beta) signaling. In *Echinococcus multilocularis* there is evidence that the host EGF is likely to affect germ cell proliferation probably through its effect on the tyrosine kinase receptor, which is a member of the family of *Echinococcus* EGF receptors (EGFR) (7, 8). Host-derived EGFs are likely to act through tyrosine kinase, which is a member of the family of EGFR of *E. multilocularis* (7). EGF can stimulate the proliferation of cells in the parasite and may interfere with the TGf-β / BMP family in metacestode infections of vertebrate hosts. It has recently been found in vitro that host TGF- β interacts with the parasite TGF- β receptors to enhance the growth of *Taenia crassiceps* metacestodes (9). In the closely related taxa in digenean trematodes, *Schistosoma*, similar patterns have also been demonstrated for tyrosine kinase receptors for insulin and EGF (10, 11).

Vitamin E is a major lipid-soluble component of biological membranes. The most important biological role of vitamin E is its antioxidant role (12, 13). Antioxidant action of vitamin E as a peroxyl radical scavenger is well known and described by many researchers (14, 15). In recent decades the non-antioxidant molecular functions of vitamin E have been widely investigated. Tocopherols and tocotrienols interact with the genes involved in oxidative stress, inflammation and apoptosis, growth, longevity, cellular signaling and gene expression in single cells such as *Rotifer philodina*, *Paramecium tetraurelia*, and *Saccharomyces cerevisiae* and nematodes e.g. *Caenorhabditis elegans* and *Turbatrix aceti* and some of insects and rodents (12). In addition, Alpha-Tocopherol has been shown to regulate some genes possibly involved in the cell proliferation functions and signal transduction (16). The deficiency of vitamin E causes alterations in the gene expression as down/up-regulation, in some of the genes involved in the stimulation of cell growth, apoptosis, protein folding and starting of cell cycle (16, 17). Alpha-tocopherol at concentrations ranging from 25 to 50 μM regulates signal transduction pathways through mechanisms other than antioxidant activity (18).

Our understanding of the nature of growth and development in *E. granulosus* has been considerably improved through the successful in vitro cultivation of the tapeworm. One of the fascinating phenomena in the biology of *E. granulosus* is its ability of growth and development either into the metacestode or the adult strobilated stages in different culture media (19). Smyth and colleagues introduced the parasite as an appropriate laboratory model for studying invertebrate biology by doing a variety of in vitro experiments on *E. granulosus* cultivation in mono- and diphasic culture systems (20). These experiments paved the way for further research and extensive studies on the parasites’ genomics, drug effects, molecular and developmental biology (21).

There is little information on alpha-tocopherol effects on the genes involving in TGF- β and EGFR expression. The purpose of the present study was to determine the effects of alpha-tocopherol on EGFR and TGF-β genes of three developmental stages of *E. granulosus* including intact invaginated protoscoleces, evaginated protoscoleces and strobilated worms.

## Materials and Methods

### Parasite materials and in vitro cultivation

Hydatid cysts were collected from Kerman municipal abattoir from naturally infected sheep liver. The cysts were immediately transferred to the laboratory, hydatid fluid containing protoscoleces was aspirated into a 50 ml tube and the parasites, as well as the entire laminated/germinal layers, were placed in sterile PBS (pH 7.4). Protoscoleces and brood capsules were washed four times in PBS, for 5–10 min. Viability of protoscoleces was determined by their flame cell motility and eosin exclusive test (22). Parasite batches with the >90% viability rates were used for in vitro cultivation.

The culture medium contained two-phases, bovine serum as the solid phase and the liquid phase, containing 260 ml CMRL1066 1X, 1.4 ml of 5% dog bile, with penicillin (100 IU/mL) and streptomycin (100 *μ*g/mL), 36 ml of 5% yeast extract, 0.341 g sodium bicarbonate (10 mM), 1.93 g HEPES (20 mM), 5.6 ml of 30% glucose, and FBS was added to a final volume of 405 ml. Then, 4 to 5 ml of the liquid phase was added to the 25-ml flasks containing solid phase (20, 23).

For evagination, intact invaginated protoscoleces in CMRL 1066 1x base medium containing 25 μl of dog bile and penicillin/streptomycin, were shaken at 90 rpm for 18 to 24 h at 37 °C in a CO2 incubator (Memmert GmbH, Germany). The protoscoleces were transferred to a diphasic culture medium under sterile conditions at 37 °C. The medium was replaced every 3–5 days with fresh culture media. The parasites were examined regularly for growth and segmentation (24).

### DNA Extraction and Genotyping

For parasite genotype identification total genomic DNA was extracted using commercial DNA extraction kits according to the manufacturer instructions (Tissue Genomic DNA Extraction Mini Kit, Favorgen Biotech Corp., Taiwan) Briefly, Micropestles were used to grind the protoscoleces. After overnight incubation with Proteinase K (10mg/ml) at 60 °C, the sample was thoroughly vortexed until the protoscoleces was lysed completely. The DNA quality was evaluated with NanoDrop 2000c Spectrophotometer (Thermo Fisher Scientific, USA) and the samples were kept at −20 °C until use. PCR amplifications were performed using *JB3* (forward) and *JB4.5* (reverse) primers (Table 1) on *cox1* gene as described earlier followed by Sanger sequencing (25, 26). The sequences were managed using BioEdit software (V.7.0.9.0), aligned with representative reference sequences and definitive identification was made by NCBI BLASTn program (https://blast.ncbi.nlm.nih.gov/Blast.cgi). The sequence data were deposited in NCBI GenBank.

**Table 1:** Primer sequences used for qPCR analysis of Epidermal Growth Factor Receptor (*EGFR*) and Transforming Growth Factor beta (*TGF- β*) genes expression and parasite genotyping based on Cytochrome C oxidase subunit 1 (*cox1*). Beta actin was used as the housekeeping reference gene

***Gene name***	***primer name***	***Sequences of forward and reverse primers***
*EGFR*	Eg-egfr	5′-CCTTGTTAGACCACCATCAC-3′ F 5′-GCGTCTTTAATCCCACTACC-3′ R
*TGF-beta*	Eg-tgf	5′-AGATGCGTGCTGAAGATG-3′ F 5′-CAGGTGTTTGAGAAGGATAAGA-3′ R
*Cox1*	JB3 JB4.5	5′-TTTTTTGGGCATCCTGAGGTTTAT-3′ F 5′-TAAAGAAAGAACATAATGAAAATG-3′ R
*Beta Actin*	ACTB	5′-TAAAGAAAGAACATAATGAAAATG-3′ F 5′-GTCGGTCGTGATCTGACTGA-3′ R

### Alpha*-tocopherol treatment of protoscoleces and strobilated worms*

In a 96-well plate 250 μg/ml alpha-tocopherol (Serva, Heidelberg, Germany) was added in each well on the 200 μl protoscoleces suspension for 36 h. For the strobilated worms, in a 12-well plate at least 10 worms were added to each well supplemented with the solid phase with the same alpha-tocopherol concentration and time. Dose-response study was carried out with different times and alpha-tocopherol concentrations. Finally of various the treatment period the protoscoleces were removed from each well and stored at −70 °C for later work. Untreated controls were considered for each experiment and all the tests were done in duplicate.

### RNA Extraction, cDNA synthesis and qPCR analyses

Total RNA for each parasite stage was extracted using Blood/Cultured Cell Total RNA Purification Mini Kit (Favorgen Biotech Corp., Taiwan) for each parasite stage in treated as well as non-treated controls. The quantity and quality of RNA were assessed using Nano Drop 2000c Spectrophotometer (Thermo Fisher Scientific, USA). cDNA synthesis was performed with the extracted RNA using a commercial kit (cDNA Synthesis Kit, YTA, Iran) according to the manufacturer’s instructions.

Using available information on the genomic structure of *E. granulosus* and the databases, GeneDB (https://www.genedb.org/), appropriate primers were designed as described in Table 1. qPCR analysis was performed for measuring relative gene expression levels using SYBR Green qPCR master Mix kit (YTA Co., Iran). According to the manufacturer's protocol, SYBR Green qPCR Mix (5 *μl*), forward and reverse primer (1*μl*, template DNA (1.5*μl*) were prepared and nuclease free water was added to a final volume of 20 *μl*. The PCR analyses were carried out in Rotor-Gene Q 6000 (QIAGEN, Hilden, Germany) with a thermal program of initial denaturation at 95 °C for 20 sec to 3 min, denaturation at 95 °C for 5 sec in 40 cycles, annealing and extension 60 °C for more than 20 sec in 40 cycles. No-template control (all but cDNA) was used in all amplification reactions. β-actin was used as the positive reference gene.

### Statistical analysis

One-way ANOVA test were used to evaluate the impact of alpha-Tocopherol on *EGFR* and *TGF- β* expressions among three developmental stages of *E. granulosus* compared to the controls. Data analysis was performed using GraphPad Prism version 8 software. 2^−ΔΔCT^ method was used for relative quantification between the stages. *P*<0.05 was considered statistically significant.

## Results

### In vitro cultivation

**Fig. 1:**
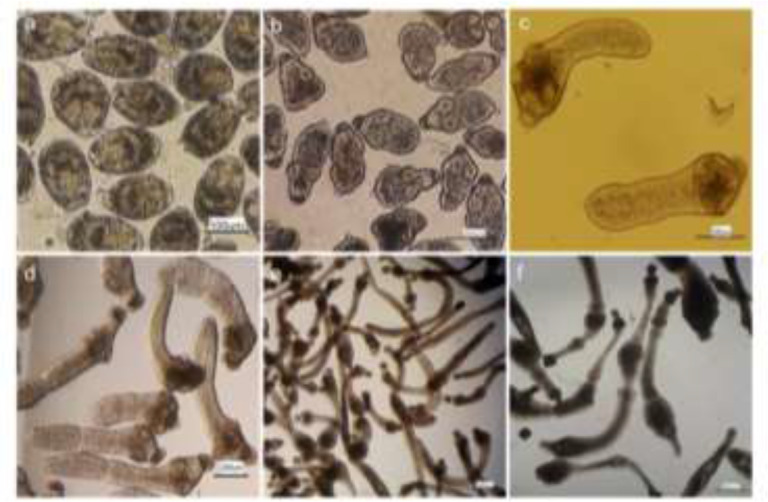
In vitro developmental stages of *Echinococcus granulosus* in diphasic culture system; (a) intact invaginated protoscoleces, (b) evaginated protoscoleces, (c) Excretory canals and bladder formation, (d) Segmented worm with one proglottid, (e) Segmented worm with two proglottids and (f) three or more proglottid worms

Intact protoscoleces were successfully developed to the segmented worms in diphasic culture media (Fig. 1). Three or more proglottid worms were obtained in about two months after protoscoleces in vitro cultivation. Different phases of invaginated protoscoleces development to the segmented worms are illustrated in Fig. 1a to Fig. 1f.

### Genotyping

DNA from all the stages was successfully extracted and the PCR amplification of the target gene produced a 460 bp cox1 fragment. After Sanger sequencing, the parasite was identified as *E. granulosus* sensu stricto G1 genotype. The sequence data obtained from the parasite was deposited in the NCBI GenBank under the accession number MG832791.

### qPCR results of TGf-β and EGFR

**Fig. 2:**
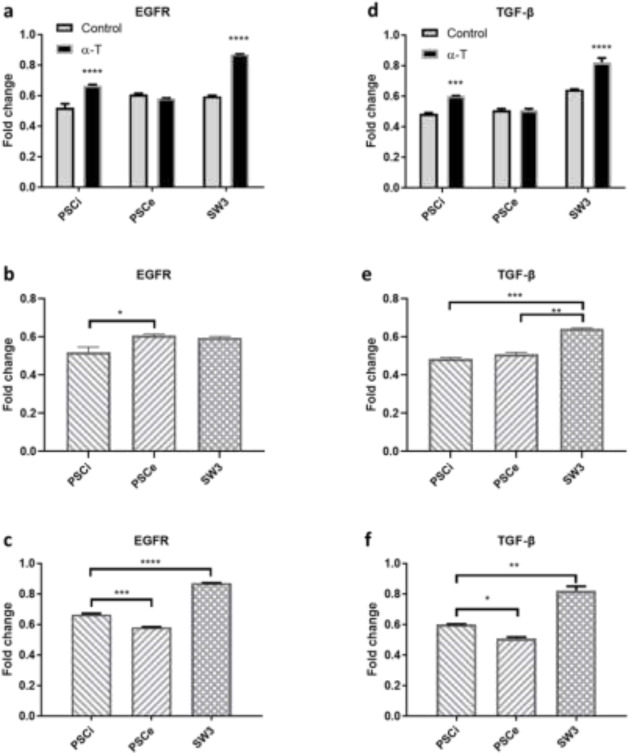
Comparative analysis of the effect of alpha-tocopherol (*α* T) (250 μg/ml for 36 h) on the expression of Epidermal Growth Factor Receptor (*EGFR*) and Transforming Growth Factor beta (*TGF- β*) in three developmental stages of *E. granulosus* (PSCi: invaginated protoscoleces; PSCe: evaginated protoscoleces; SW3: segmented worms with three proglottids). Panel a–f: Expression changes of *EGFR* (a) and *TGF- β* (d) induced by *α*T compared with no-treatment controls. Comparison of expression changes of EGFR and *TGF- β* in the three developmental stages in untreated (b and e) and treated groups (c and f). *P<0.05, **P<0.01, and ***P<0.001, ****P<0.0001

Alpha-tocopherol treatment (250 *μ* g/ml alpha-tocopherol for 36 h) resulted in a significant increase in the expression of *EGFR* in the invaginated protoscoleces and segmented worms compared to the controls. However, there was no significant difference in evaginated protoscoleces between alpha-tocopherol and control groups (Fig. 2a). As shown in Fig. 2d, the results indicated a significant increase in the expression of *TGF-β* gene in the invaginated protoscoleces and segmented worms compared to the control groups. Similar to *EGFR*, no significant difference was found in the *TGF- β* expression in evaginated protoscoleces compared to the controls (Fig. 2d).

Comparing non-treated controls, evaginated protoscoleces presented the highest *EGFR* expression in comparison with invaginated protoscoleces and segmented worms. This was not the case for *TGF- β* in which the gene expression in the segmented worms was significantly higher than invaginated and evaginated protoscoleces (fig. 2b and 2e). Regarding the parasite *EGFR*/*TGF- β* genes response to alpha-tocopherol treatments, the segmented worms presented the highest expressions of both genes compared to the other two stages (Fig. 2c and 2f).

## Discussion

Evidence indicated the effect of different cytokines and hormones on EGF and TGF- β signaling in a wide range of eukaryotic organisms including insects, nematodes and mammals. In the present study in different developmental stages of *E. granulosus*, we explored the effects of alpha-tocopherol on the expression of two important signaling molecules that control essential functions related to growth and development in many cell types, i.e. the receptor tyrosine kinase, EGFR and TGF- β as a multifunctional peptide. Several cellular/molecular functions have been attributed to vitamin E including alpha-tocopherol as one of the main compounds in vitamin E family.

The most well-known biological role of alpha-tocopherol is its antioxidant activity and radical scavenging. However, non-antioxidant functions of alpha-tocopherol have recently been demonstrated in eukaryotic organisms including different protozoan and helminth parasites (27). Several studies have investigated the interactions of vitamin E compounds with different genes involved in the basic cellular phenomena such as cellular signaling, growth, oxidative stress, longevity, inflammation and apoptosis (12). However, there are not much data on the role of alpha-tocopherol in the cellular and molecular biology of platyhelminths.

Findings of the present study indicated the significant effects of alpha-tocopherol on *EGFR* and *TGF- β* in certain developmental phases of *E. granulosus*. Pronounced over-expression of both *EGFR* and *TGF- β* were observed in the strobilated worms as well as the invaginated protoscoleces. Nevertheless, the evaginated protoscoleces of *E. granulosus* did not show any significant changes of the genes expression after alpha-tocopherol treatment. This is following several other studies confirming the inductive role of alpha-tocopherol on the expression of *EGFR* and *TGF- β* (28–30). The effect of alpha-tocopherol on the cell regulation and expression of several genes including *TGF- β* in human cell lines has been investigated. A significant overexpression of *TGF- β* has been demonstrated in human smooth muscle cells (SMCs) and fibroblasts (31). In another study EGFR signaling pathway in *E. multilocularis* promoted germinative cell proliferation upon in vitro addition of 100 ng/ml recombinant human EGF and therefore EGFR/ERK signaling pathway has been suggested as a potential therapeutic target for the treatment of alveolar echinococcosis (7). As our findings indicated that alpha-tocopherol increased the expression of EGFR gene in *E. granulosus*, use of both alpha-tocopherol and EGF have a synergistic effect on *EGFR* gene overexpression. Further in vitro and in vivo studies on this issue are required to elucidate the nature of the parasite response to vitamin E.

Considerable homology of cell signaling of receptor tyrosine- and receptor serine/threonine kinases have been shown in *Echinococcus* and its hosts (32), therefore the same alpha-tocopherol-induced overexpression processes could be explained for *E. granulosus* as for human cell lines.

Our results indicated that while alpha-tocopherol exerted no significant effect on *EGFR* expression in the evaginated protoscoleces, highly significant difference in *EGFR* expression was observed in the in vitro segmented worms (*P*<0.0001, Fig. 2a). Similar findings were obtained for *TGF- β* when comparing evaginated protoscoleces and the segmented worms with their no-treatment controls (*P*<0.0001, Fig. 2d).

It has been demonstrated in several parasitic organisms including *Plasmodium* and *Schistosoma* species that the parasites use vitamin E to avoid oxidative stress (33). As was mentioned earlier the antioxidant function of vitamin E compounds could explain the findings of the present study. Increasing amounts of antioxidant levels led to a decrease of free radicals and this can protect the organisms against cytotoxic actions of free radicals (34).

Comparing the expression of the genes studied in various stages, a general pattern of increasing expression of *EGFR* and *TGF- β* was documented during growth and segmentation of *E. granulosus* in vitro (Figs. 2b and 2e). Similar findings have been reported with other genes such as *SmadD*, *HoxB7* and *notch* genes (35). This is a meaningful finding particularly for *SmadD*, as we know this gene is a downstream factor of TGF- β signaling (32). The genes involving in TGF- β and/or EGFR pathways are known to have a role in the growth, development, cell proliferation and differentiation of eukaryotic organisms (36).

## Conclusion

To our knowledge this study is the first examination of alpha-tocopherol effects on *E. granulosus* in an in vitro culture system. Administration of alpha-tocopherol to different stages of *E. granulosus* significantly enhanced *EGFR* and *TGF- β* expression. Overexpression of the genes could, in turn, enhance growth factor effects and improves living conditions of the parasite.
